# Structural Basis of Inhibition and Desensitization in Heteromeric Kainate Receptors

**DOI:** 10.64898/2025.12.02.691905

**Published:** 2025-12-04

**Authors:** Changping Zhou, Guadalupe Segura-Covarrubias, Ashlee M’Kay Hoffman, Nami Tajima

## Abstract

Kainate receptors (KARs) mediate excitatory synaptic transmission and regulate neurotransmitter release. In the central nervous system, KARs predominantly exist as heterotetramers comprising low-affinity (GluK1–3) and high-affinity (GluK4–5) subunits, with GluK2/GluK5 being the most abundant. To elucidate their conformational transitions, we determined electron cryo-microscopy (cryo-EM) structures of GluK2/GluK5 KARs in multiple functional states. The apo structure reveals compact packing with extensive intersubunit interactions between the ligand-binding domains (LBDs), beyond conserved D1–D1 contacts. Similarly, the glutamate-bound structure exhibits enhanced packing that stabilizes the desensitized conformation through increased intersubunit contacts relative to homomeric KARs, indicating that heterotetramers are conformationally less dynamic. To investigate subtype-specific inhibition, we engineered GluK2 and GluK5 mutants with altered affinities for the antagonist UBP310. Structural analysis of these mutants reveals distinct UBP310 binding modes on GluK2 versus GluK5 subunits. Furthermore, we demonstrate that targeting GluK5 is more effective than targeting GluK2; stabilizing GluK5 locks the receptor in a pore-occluded conformation, whereas antagonizing GluK2 leaves considerable physical space above the pore. These findings provide a structural framework for understanding the distinct contributions of GluK2 and GluK5 subunits to KAR function and highlight new strategies for developing subunit-specific therapeutics.

## Full Text Availability

The license terms selected by the author(s) for this preprint version do not permit archiving in PMC. The full text is available from the preprint server.

